# An LDPC Decoder Architecture for Wireless Sensor Network Applications

**DOI:** 10.3390/s120201529

**Published:** 2012-02-06

**Authors:** Andrea Dario Giancarlo Biroli, Maurizio Martina, Guido Masera

**Affiliations:** Dipartimento di Elettronica e Telecomunicazioni, Politecnico di Torino, Corso Duca degli Abruzzi 24, Torino 10129, Italy; E-Mails: andreadariogiancarlo.biroli@studenti.polito.it (A.D.G.B.); guido.masera@polito.it (G.M.)

**Keywords:** LDPC decoder architecture, wireless sensor networks, power consumption

## Abstract

The pervasive use of wireless sensors in a growing spectrum of human activities reinforces the need for devices with low energy dissipation. In this work, coded communication between a couple of wireless sensor devices is considered as a method to reduce the dissipated energy per transmitted bit with respect to uncoded communication. Different Low Density Parity Check (LDPC) codes are considered to this purpose and post layout results are shown for a low-area low-energy decoder, which offers percentage energy savings with respect to the uncoded solution in the range of 40%–80%, depending on considered environment, distance and bit error rate.

## 1. Introduction

Wireless Sensor Networks (WSN) have gained growing research interest in the last years. The possibility to monitor different physical quantities even in dangerous and hard-to-reach areas has found applications in several fields, including medical, industrial and surveillance environments [1]. WSNs are made of small nodes, where each node often relies on small size and light weight batteries. As a consequence, both energy consumption and area occupation are important aspects in the design of nodes. Although nodes feature a limited energy budget, they embody not only sensing but also computational and transmit/receive circuits. Thus, energy consumption issues are critical and ought to be minimized at every design level. As an example in [2] several system level techniques, including modulation, Media Access Control (MAC) protocols and channel coding techniques are analyzed to achieve energy efficiency in WSNs.

In [3] it is shown that in WSNs the transmission energy can be lowered accepting to receive error-affected data. In this case the receiver should embed error correction strategies to recover the original data. In particular, the amount of energy spent to perform error correction should be significantly lower than the energy saved at the transmitter side. As an example, in [4,5] an energy efficient error correction scheme for WSNs is proposed. In particular, in [5] the physical layer of the IEEE 802.15.4 standard [6] is augmented introducing interleaving and forward error correction. In [2,3] several classes of codes are investigated, including Reed–Solomon codes, convolutional codes, turbo codes and Low-Density-Parity-Check (LDPC) codes [7,8]. Experimental results in [3] show that LDPC codes are good candidates for WSN applications as they feature a significant coding gain as compared with other codes. However, they consume about one order of magnitude more than simpler codes as the extended Hamming ones. Most of previous works proposing error correction codes for WSNs assume that networks contain at least two classes of nodes: sensing nodes and central nodes. Sensing nodes feature lower computational capabilities and available energy than central nodes. Thus, sensing nodes send coded information to a central node which performs the decoding operations. On the contrary, this work investigates homogeneous WSNs where each node can both transmit and receive coded information. A similar idea is proposed in [9] with focus on turbo codes. In particular, in [9] it is shown that the energy consumption of homogeneous WSN is reduced by about 70% resorting to turbo codes. In this work we show that even higher energy saving and smaller area can be achieved with LDPC codes. In particular, this work shows that small block length LDPC codes are adequate for typical throughput and data transmission requirements of WSNs.

The paper is structured as follows: Section 2 deals with LDPC coding and decoding algorithms whereas Section 3 concentrates on modeling the WSN environment. Section 4 details the proposed LDPC decoder architecture and Section 5 shows the experimental results. Finally, in Section 6 conclusions are drawn.

## 2. Coding and Decoding Algorithms for LDPC Codes

LDPC codes are a class of linear block codes, characterized by a very sparse *M* × *N* parity-check matrix **H** where valid codewords *x* satisfy **H** · (*x*)′ = 0 and (·)′ represents the transposition operator. Each LDPC code can be represented as a bipartite graph, known as Tanner Graph [10], containing two sets of nodes: Variable Nodes (VNs) and Check Nodes (CNs). VNs are associated to the *N* bits of the codeword, whereas CNs correspond to the *M* parity-check constraints. Edges in the graph correspond to ones in the **H** and most of decoding algorithms imply the exchange of information along the edges of the Tanner graph. The most common algorithm to decode LDPC codes is the *Belief Propagation* (BP) algorithm. The VNs receive the intrinsic information λ (likelihood functions *i.e.*, probabilities) from the channel and update it depending on the results of the parity check equations computed at the CNs. This process is iterated several times until either the maximum number of iterations is reached, or a convergence criterion is met. This criterion may be that a codeword was successfully decoded.

There are two main scheduling schemes for the BP [11]: two-phase scheduling and layered scheduling [12]. The latter nearly doubles the convergence speed as compared to two-phase scheduling. In a layered decoder, parity-check constraints are grouped in layers, each of which is associated to a component code. Then, layers are decoded in sequence by propagating extrinsic information from one layer to the following one [12]. When all layers have been decoded, one iteration is complete and the overall process can be iteratively repeated up to the desired level of reliability.

Let *S_j_* represent the Log-Likelihood-Ratio (LLR) of the bit in column *j* of **H**. Bit LLR *S_j_* is initialized to the corresponding received soft value. Then, for each parity constraints *m* in a given layer, the following operations are executed:

(1) $$Q_{\mathrm{mj}}=S_{j}^{\left(\text{old}\right)}-R_{\mathrm{mj}}^{\left(\text{old}\right)}$$

(2) $$A_{\mathrm{mj}}=\sum_{n\in {𝒩}_{m},n\neq j}\Psi \left(Q_{\mathrm{mn}}\right)$$

(3) $$s_{\mathrm{mj}}=\prod_{n\in {𝒩}_{m},n\neq j}\text{sgn}\left(Q_{\mathrm{mn}}\right)$$

(4) $$R_{\mathrm{mj}}^{\left(\text{new}\right)}=-s_{\mathrm{mj}}\cdot \Psi \left(A_{\mathrm{mj}}\right)$$

(5) $$S_{j}^{\left(\text{new}\right)}=Q_{\mathrm{mj}}+R_{\mathrm{mj}}^{\left(\text{new}\right)}$$

$S_{j}^{\left(\text{old}\right)}$ is the extrinsic information received from the previous layer and updated in Equation (5) to be propagated to the succeeding layer. Term $R_{\mathrm{mj}}^{\left(\text{old}\right)}$, pertaining to element (*m,j*) of **H**, is used to compute Equation (1); the same amount is then updated in Equation (4), $R_{\mathrm{mj}}^{\left(\text{new}\right)}$, and stored to be used again in the following iteration. In Equations (2) and (3) *𝒩_m_* is the set of all bit indices that are connected to parity constraint *m*.

Unfortunately, the computation of Equations (2) and (4) is complex, as Ψ (·) is a non-linear function. According to [13], Equation (2) can be simplified with a limited Bit-Error-Rate (BER) performance loss as

(6) $$R_{\mathrm{mj}}^{\mathrm{new}}\approx -{s^{\prime }}_{\mathrm{mj}}\cdot \underset{t\in {𝒩}_{m}\backslash j}{\text{min}}\{|Q_{t_{j}}|\}$$

usually referred to as *normalized-min-sum* approximation, where *s′_mj_* = σ · *s_mj_* and σ ≤ 1. For further details the reader can refer to [8,10].

A key concern in the design of high throughput LDPC code decoders comes from the communication structure that must be allocated to support message passing among VNs and CNs. Three approaches can be followed in the high level organization of the decoder:

1. Fully Parallel Architectures (FPA): separate processing units are allocated for each VN and CN and all messages are passed in parallel along dedicated routes.
2. Partially Parallel Architectures (PPA): more processing units work in parallel, serving all VNs and CNs within a number of cycles; suitable organization and hardware support is required to exchange messages.
3. Serial architectures (SA): a single processing instance is allocated for both VN and CN computations and nodes are served sequentially; messages are exchanged by means of a unique memory.

The first approach leads to very high throughput, large implementation cost and severe congestion problems in the routing of interconnects [14]. For these reasons it is not adopted in practical implementations. The partially parallel architecture requires a large bandwidth between processing units and memories where messages are stored. Moreover, special attention is necessary to avoid collisions in the memory access [15]. However, the partially parallel organization allows to precisely tune the wanted degree of parallelism with respect to the addressed throughput and it was proved to be the best solution for the implementation of efficient decoders [15–19]. The serial approach leads to low cost and low power implementations and it also offers a high level of flexibility with respect to the supported code. However serial architectures did not receive much attention, due to the fact that the sequential processing does not achieve large throughput. This solution is particularly suitable for software implementations on Digital Signal Processors [20]. As throughput requirements in WSN applications are usually much lower than in wireless communications, the serial approach appears as the best solution to implement low cost and low energy decoding in a sensor node.

## 3. Wireless Sensor Network Environment and Modeling

Required throughput and energy budget are important parameters to model the environment of a WSN. Although the throughput depends on the application, several recent works [21–24] as well as off-the-shelf products for the IEEE 802.15.4 standard target a throughput *T* of 250 kb/s. According to [3] the amount of energy per bit saved due to the use of a correcting code (Δ*E*) can be expressed as

(7) $$\Delta E=E_{\mathrm{TX},U}-E_{\mathrm{TX},C}-E_{\mathrm{enc}}-E_{\mathrm{dec}}$$

where *E_TX,U_* and *E_TX,C_* are the amounts of energy per information bit spent to transmit one bit in an uncoded and coded system respectively. *E_enc_* and *E_dec_* are the amounts of energy per bit spent by the LDPC encoder and decoder. Assuming a Binary-Phase-Shift-Keying (BPSK) modulation, each *E* term in Equation (7) can be written as a function of the power consumption *P* and the throughput *T* of the corresponding task. For a fair comparison we assume that the throughput sustained by the transmitter is the same for both the uncoded and coded case. As a consequence, Equation (7) can be rewritten as

(8) $$\Delta E=\frac{P_{\mathrm{TX},U}-P_{\mathrm{TX},C}-P_{\mathrm{enc}}-P_{\mathrm{dec}}}{T}$$

However, as shown in [25] and [26] the complexity and the power consumption of LDPC encoding is negligible with respect to decoding. As a consequence, in the following the *P_enc_* term will be neglected. Moreover, as highlighted in [3], each *P_TX_* term can be written as a function of the path loss *A*(*d*) at a given distance *d*, the thermal noise *N*_0_ · *B* (where *B* is the signal bandwidth and *N*_0_ is the noise power spectral density), the Signal-to-Noise-Ratio (SNR) at the receiver and the receiver noise figure *F* :

(9) $$P_{\mathrm{TX}}=A(d)\cdot N_{0}\cdot B\cdot 10^{(\text{SNR}+F)/10}$$

According to [27],

(10) $$A(d)={\left(\frac{4\pi }{\lambda }\right)}^{2}\cdot d^{n}$$

where λ is the wavelength of the corresponding carrier frequency *f* and *n* is the path loss exponent, where *n* = 2 and *n* = 4 are good approximations for free space and dense environment propagations respectively. Assuming the same *A*(*d*) and *F* values for both uncoded and coded systems, Equation (8) can be rewritten as

(11) $$\Delta E=\frac{A(d)\cdot N_{0}\cdot B\cdot 10^{F/10}\cdot \left(10^{{\text{SNR}}_{U}/10}-10^{{\text{SNR}}_{C}/10}\right)-P_{\mathrm{dec}}}{T}$$

where SNR*_U_* and SNR*_C_* are the SNR at the receiver in the uncoded and coded systems respectively. Thus, given the curves representing the BER of one system as a function of the SNR, we obtain for each BER value the amounts SNR*_U_* and SNR*_C_* with SNR*_G_* = SNR*_U_* − SNR*_C_* representing the SNR gain achieved using error correction. So Equation (11) can be rewritten as

(12) $$\Delta E=\frac{A(d)\cdot N_{0}\cdot B\cdot 10^{({\text{SNR}}_{U}+F)/10}\cdot \left(1-10^{-{\text{SNR}}_{G}/10}\right)-P_{\mathrm{dec}}}{T}$$

The expression obtained in Equation (12) will be used in Section 5 to show the effectiveness of the proposed LDPC architecture.

## 4. LDPC Decoder Architecture Design

LDPC codes are known to nearly achieve the Shannon limit when the block of data is very large (*N* → ∞) [10]. However, in WSN applications the amount of bits exchanged by nodes is limited, leading to small *N* values. Nevertheless, in [28,29] it is shown that LDPC codes can achieve excellent performance even when *N* is small. In this work, we analyze the minimum *N* LDPC code from the IEEE 802.16e standard [30], which corresponds to *N* = 576 coded bits and *K* = *R · N* = 288 uncoded bits (*R* = 0.5). Moreover, we considered the two best performing regular codes with *N* = 96 and *N* = 204 (*K* = 48, *K* = 102) respectively, taken from MacKay database [31] and referred to as 96.33.966 and 204.33.484 (*R* = 0.5 for both).

**Figure 1.**
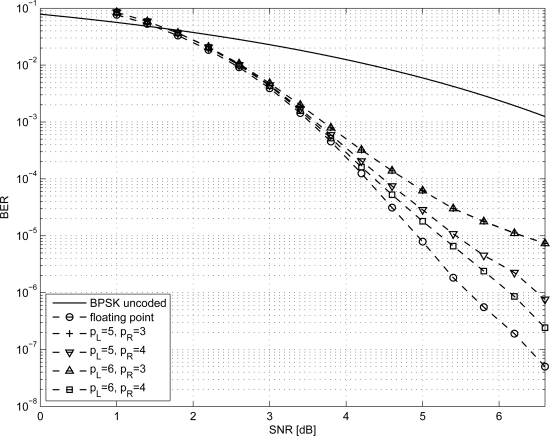
BER performance of the *N* = 96 LDPC code.

**Figure 2.**
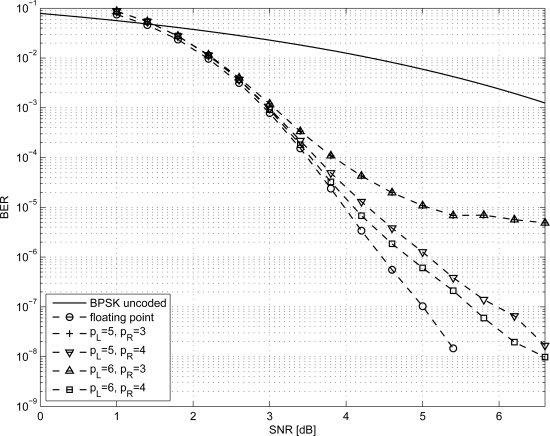
BER performance of the *N* = 204 LDPC code.

**Figure 3.**
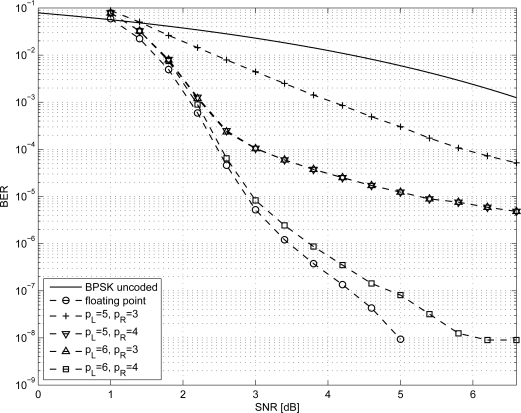
BER performance of the *N* = 576 LDPC code.

In order to size the LDPC decoder architecture, finite precision analysis ought to be performed. Given that *p_S_* and *p_R_* are the number of bits to represent *S_j_* and *R_mj_* metrics respectively, as in Equations (1–6), simulations have been carried out for *p_S_* ∈ {5, 6} and *p_R_* ∈ {3, 4}; normalized-min-sum approximation with σ = 0.875 has been employed. The performance of the three considered codes are shown in Figures 1–3 both in the floating point and fixed point cases together with the performance of the corresponding uncoded system. Furthermore, it has been observed that targeting a BER of 10^−4^ as in [3,9] and imposing a maximum of ten iterations (*I* = 10), the performance loss is negligible.

Due to the low throughput required, we assume that a fully serial processor architecture, which executes the decoding algorithm on one CN at the time, is a reasonable solution. In this case the throughput sustained by the architecture, defined as the number of decoded bits over the decoding time, is

(13) $$T=\frac{K\cdot f_{\mathrm{clk}}}{M\cdot I\cdot d_{c}^{\text{max}}+D}=\frac{K\cdot f_{\mathrm{clk}}}{\frac{1-R}{R}\cdot K\cdot I\cdot d_{c}^{\text{max}}+D}$$

where *f_clk_* is the decoder clock frequency, *I* is the maximum number of iterations, $d_{c}^{\text{max}}$ is the maximum degree of a CN, *i.e.*, the maximum number of edges on a CN and *D* is the latency of the architecture. It is worth noting that Equation (13) can be adapted to parallel and partially parallel architectures by substituting *M* with *M/W* where *W* is the number of rows (in **H**) processed in one clock cycle. The latency *D* in Equation (13) can be minimized avoiding idle cycles between iterations, so that $D=d_{c}^{\text{max}}$. Thus, the throughput can be approximated as

(14) $$T=\frac{K\cdot f_{\mathrm{clk}}}{\left(\frac{1-R}{R}\cdot K\cdot I+1\right)\cdot d_{c}^{\text{max}}}\approx \frac{R\cdot f_{\mathrm{clk}}}{(1-R)\cdot I\cdot d_{c}^{\text{max}}}$$

**Figure 4.**
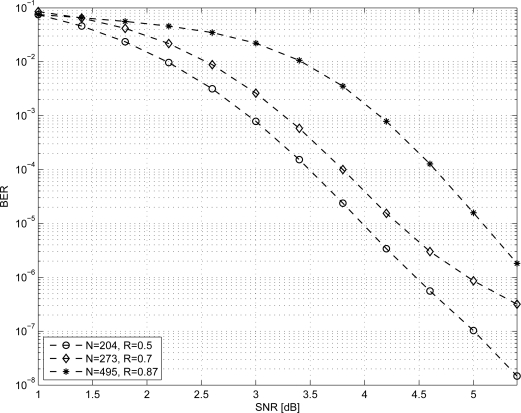
BER performance of the (*N* = 204, *R* = 0.5), (*N* = 273, *R* = 0.7) and (*N* = 495, *R* = 0.87) LDPC codes.

**Figure 5.**
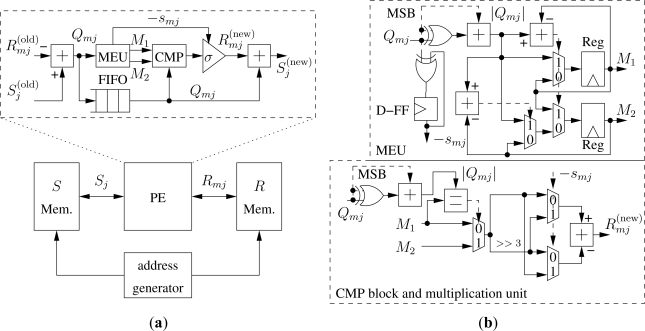
Proposed decoder architecture: (**a**) general structure and PE detail; (**b**) MEU, CMP block and multiplication unit block schemes.

As it can be observed, the throughput increases with *R* so low-rate codes are a conservative choice to achieve the target throughput. Moreover, if we fix *N* we observe that increasing the rate has the effect of reducing the BER performance of the code. Thus, we considered the *N* = 204, *R* = 0.5 code and tried to increase both *N* and *R*. From MacKay database [31] we considered the following two high-rate codes where *N* > 204: *N* = 273, *R* = 0.7 and *N* = 495, *R* = 0.87 referred to as 273.82.3.353 and 495.62.3.2915 respectively. As shown in Figure 4 the BER performance of both codes is lower than the one obtained for *N* = 204, *R* = 0.5. Furthermore, codes with *N* > 204 require a larger amount of memory than the *N* = 204, *R* = 0.5 code. From this analysis we infer that for the most complex code among the ones considered in this work, *i.e.*, $d_{c}^{\text{max}}=7$ for the IEEE 802.16e *N* = 576, *R* = 0.5 code, and given the target throughput *T* = 250 kb/s and *I* = 10, Equation (14) leads to *f_clk_* ≥ 17.5 MHz. In this work we fix *f_clk_* = 20 MHz as a conservative value. Thus, the proposed architecture, inspired by the data-path of the solution proposed in [32], is made of four blocks as shown in the bottom part of Figure 5(a): a processing element (PE) devoted to implement the computation described in Equations (1–6) with the normalized-min-sum approximation; *S* and *R* memories, where *S_j_* and *R_mj_* metrics are stored; and an address generator. As depicted in the upper part of Figure 5(a) the PE contains: (i) a subtractor to compute *Q_mj_* Equation (1), (ii) a Minimum-Extractor-Unit (MEU), a compare block (CMP) and a multiplication by ±σ required to compute *s_mj_* Equation (3) and $R_{\mathrm{mj}}^{\left(\text{new}\right)}$ with the normalized-min-sum approximation Equation (6), (iii) a synchronization FIFO with $d_{c}^{\text{max}}$ locations, (iv) an adder to compute $S_{j}^{\left(\text{new}\right)}$ Equation (5).

The MEU, detailed in the upper part of Figure 5(b) is made of two parts. The first one computes −*s_mj_* xoring the sign of *Q_mn_* values, *i.e.*, the most significant bit (MSB) of *Q_mn_*, and saving the result in a D-Flip-Flop (D-FF). The second part computes the absolute value of *Q*_*tj*_. Then, since the min function in Equation (6) is on *𝒩_m_\j*, the MEU finds the first two minimum values among the possible *𝒩_m_* leaving to the CMP block to exclude the *j*-th one. The first two minimum values (*M*_1_ and *M*_2_) are obtained by the means of two subtractors, three multiplexer and two registers that implement Algorithm 1, where MPV is the Maximum Positive Value.

**Algorithm 1:** Algorithm to find the first two minimum values

**Require:***M*_1_ ← MPV and *M*_2_ ← MPV	
1:	**for***t* ∈ *𝒩_m_***do**
2:	**if** |*Q*_*tj*_| < *M*_1_**then**
3:	*M*_2_ ← *M*_1_
4:	*M*_1_ ← |*Q*_*tj*_|
5:	**else if** |*Q*_*tj*_| < *M*_2_**then**
6:	*M*_2_ ← |*Q*_*tj*_|
7:	**end if**
8:	**end for**

The CMP block and the multiplication unit are shown in the bottom part of Figure 5(b). The CMP block compares |*Q_mj_*| with *M*_1_. If they are equal, *M*_2_ is passed to the multiplication unit. The multiplication unit does not contain a real multiplier as σ = 0.875 = 1 − 1/8 requires only a subtractor and a hard-wired three-bit right shift (>> 3). In order to take into account the −*s_mj_* term, two multiplexers, driven by −*s_mj_* are added to obtain $R_{\mathrm{mj}}^{\left(\text{new}\right)}$ as in Equation (6).

## 5. Experimental Results

The proposed architecture has been described using VHDL language. The complete design flow, including synthesis, place and route has been performed with Synopsys Design Compiler and Cadence Encounter on a 90 nm CMOS standard cell technology with 9 levels of metal and supply voltage equal to 1 V. Post place and route simulations was run to obtain accurate capacitances and switching activities [33], which are necessary for estimating the power consumption. Area and power consumption results for the three codes analyzed in Section 4 with *p_S_* ∈ {5, 6}, *p_R_* ∈ {3, 4} and *f_clk_* = 20 MHz are shown in Table 1.

It is worth noting that it is difficult to make a fair comparison of the proposed architectures with other solutions proposed in the literature because the target applications are different. However, for the sake of completeness in Table 2 several LDPC decoder architectures are compared with the most area demanding and power consuming solution among the proposed ones (*N* = 576, *p_S_* = 6, *p_R_* = 4, last row of Table 1).

As it can be observed, most solutions proposed in the literature address partially parallel architectures designed for wireless communications and broadcasting applications. As a consequence, they are sized to obtain throughput of hundreds of Mb/s or even Gb/s with large blocks of data. On the contrary, the proposed serial architecture is specifically tailored for WSN applications where throughput and block length are much smaller, we assume here *T* ≤ 250 kb/s and *N* ≤ 576. Since the considered architectures have been designed on different technologies, we scale them all to the 90 nm technology node (*A*_90_) for the sake of fairness. The scaling is obtained multiplying the area (fifth column in Table 2) by (*𝒡*/90)^2^, where *𝒡* is the feature size shown in the fourth column of Table 2. As expected, the proposed architecture is about one order of magnitude smaller than the other ones (fifth and sixth columns in Table 2). On the contrary, partially parallel architectures consume less energy per bit and energy per bit per iteration than serial solutions (eleventh and twelfth columns in Table 2). Assuming that area and energy consumption are the most important metrics to choose a decoder architecture for WSN applications, we introduce two figures of merit. The first one is the normalized area Φ*_A_*(*k*) = *A*_90_(*k*)/ min*_k_*{*A*_90_(*k*)} where *A*_90_(*k*) is the area of the *k*-th architecture scaled to the 90 nm technology node. The second one is the normalized energy per bit per iteration Φ*_E_*(*k*) = *E_I_*(*k*)/ min*_k_*{*E_I_*(*K*)}. These two figures of merit represent how far an architecture is from the minimum area and minimum energy per bit per iterations ones respectively. Assuming that Φ*_A_* and Φ*_E_* are equally important, their product shows which architecture is more suited for WSN applications among the compared ones. As shown in the last column of Table 2 the proposed architecture is the one with minimum Φ = Φ*_A_* · Φ*_E_*. It is worth noting that as shown in the last two rows of Table 2 the proposed architecture shows better area and energy figures than the recently proposed turbo decoder architecture for WSN applications described in [9].

As highlighted in [36], several standards have been proposed for WSNs. It can be interestingly noted that most of them rely on the physical layer of the IEEE 802.15.4 standard. Thus, to evaluate the gain of the proposed architecture in a WSN environment we assume typical parameters taken from the IEEE 802.15.4 standard, namely *f* = 2.4 GHz and *B* = 80 MHz and we fix *d* = 50 m. Moreover, employing an ultra-low-power low-noise-amplifier, as the one proposed in [37], we can fix *F* = 3.8 dB.

**Figure 6.**
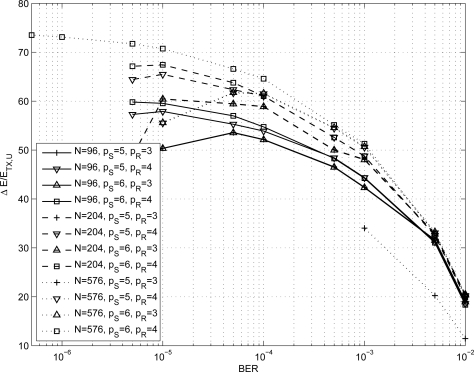
Percentage of energy per bit saved as a function of the BER for *n* = 3.

**Figure 7.**
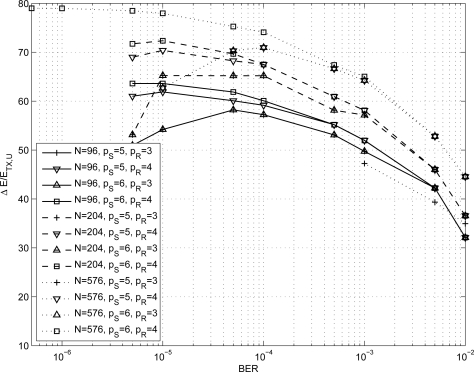
Percentage of energy per bit saved as a function of the BER for *n* = 4.

In the following we investigate the energy saving obtained for a path loss exponent equal to three and four respectively, to model either typical indoor environments and outdoor urban/suburban foliated areas [38] or dense outdoor urban environments [39]. From Equation (12) the energy per bit required by an uncoded system ranges from tens of nJ/bit to few *μ*J/bit depending on the considered path loss exponent value. As a consequence, to obtain a more significant information we compute the percentage of saved energy per bit with respect to the energy per bit of an uncoded system (Δ*E*/*E_TX,U_*) as a function of the BER. The percentage of saved energy as function of the BER for all the results shown in Table 1 is depicted in Figures 6 and 7 for *n* = 3 and *n* = 4 respectively.

As it can be observed, both for *n* = 3 and *n* = 4 at a BER of 10^−4^ the percentage of saved energy is more than the 50% and, in the best case, it achieves the 80%. It is worth pointing out that when a code reaches the error floor region, the percentage of saved energy is maximum and then it decreases. Thus, the best energy saving performance is achieved in the waterfall region of the code.

## 6. Conclusions

Notwithstanding continuous progresses in the capacity of batteries, minimizing the energy dissipation still is one of the key objectives in the design of most sensor devices. In particular, transmission energy is a relevant component of the overall energy budget of a wireless sensor. This paper explores the use of LDPC codes to protect sent information against channel errors, thus allowing for a lower transmission energy. The energy that is saved at the transmission side depends on the coding gain of the selected code: more powerful the code, larger the saved energy. However a decoder is required at the receiver side to reconstruct the original information. The node to node communication throughput is low in wireless sensor applications and this enables the design of a fully serial decoding architecture, with limited implementation complexity and extremely low dissipated power. The additional energy consumed by the decoder has been evaluated by means of logical synthesis and layout generation. Final results prove that percentage saving as high as 80% can be achieved with the coded approach with respect to the usual uncoded transmission.

## Figures and Tables

**Table 1.** Post place and route area and power consumption of the proposed architecture.

***N***	***p****_S_*	***p****_R_*	**Area [*μ*m^2^]**	***P****_dec_***[*μ*W]**
96	5	3	66,046	359
96	5	4	67,994	373
96	6	3	67,752	363
96	6	4	69,720	379
204	5	3	86,165	445
204	5	4	88,670	458
204	6	3	88,283	448
204	6	4	90,613	459
576	5	3	125,257	648
576	5	4	131,681	670
576	6	3	128,146	663
576	6	4	133,934	674

**Table 2.** Comparison of different architectures.

**Reference**	**Arch.**	***N***	**Tech. [nm]**	**Area [mm**^2^**]**	***A*_90_ [mm^2^]**	***f****_clk_***[MHz]**	***T* [Mb/s]**	***P****_dec_***[mW]**	***I***	***E* [pJ/b]**	***E_I_* [pJ/b/it]**	**Φ**
[14]	FPA	1,024	160	52.5	16.6	64	1,000	690	64	690	11	404
[18]	PPA	64,800	90	13.1	13.1	270	180	853	-	4,740	-	-
[19]	PPA	2,304	130	4.8	2.3	214	955	397	10	416	42	141
[32]	PPA	64,800	90	4.1	4.1	300	90	-	30	-	-	-
[34]	PPA	1,944	130	7.4	3.5	111	250	76	8	304	38	197
[35]	PPA	2,048	65	7.15	13.7	300	6,680	1,030	8	154	19	95

[9]	SA ^(a)^	6,144	90	0.35	0.35	333	1.03	4.17	5	4,049	810	198
This	SA	576	90	0.13	0.13	20	0.25	0.67	10	2,696	270	25

(a) Serial turbo decoder architecture for WSN applications.
